# Altered brain activation during reward anticipation in bipolar disorder

**DOI:** 10.1038/s41398-022-02075-w

**Published:** 2022-07-28

**Authors:** Xipeng Long, Xiuli Wang, Fangfang Tian, Yuan Cao, Hongsheng Xie, Zhiyun Jia

**Affiliations:** 1 Department of Nuclear Medicine, West China Hospital of Sichuan University, No. 37 GuoXue Xiang, Chengdu, Sichuan PR China; 2 Department of Clinical Psychiatry, the Fourth People’s Hospital of Chengdu, Chengdu, Sichuan PR China; 3 Department of Nuclear Medicine, the First Affiliated Hospital of Chongqing Medical University, Chongqing, PR China

**Keywords:** Bipolar disorder, Human behaviour

## Abstract

Although altered reward sensitivity has been observed in individuals with bipolar disorder (BD), the brain function findings related to reward processing remain unexplored and inconsistent. This meta-analysis aimed to identify brain activation alterations underlying reward anticipation in BD. A systematic literature research was conducted to identify fMRI studies of reward-relevant tasks performed by BD individuals. Using Anisotropic Effect Size Signed Differential Mapping, whole-brain and ROI of the ventral striatum (VS) coordinate-based meta-analyses were performed to explore brain regions showing anomalous activation in individuals with BD compared to healthy controls (HC), respectively. A total of 21 studies were identified in the meta-analysis, 15 of which were included in the whole-brain meta-analysis and 17 in the ROI meta-analysis. The whole-brain meta-analysis revealed hypoactivation in the bilateral angular gyrus and right inferior frontal gyrus during reward anticipation in individuals with BD compared to HC. No significant activation differences were observed in bilateral VS between two groups by whole-brain or ROI-based meta-analysis. Individuals with BD type I and individuals with euthymic BD showed altered activation in prefrontal, angular, fusiform, middle occipital gyrus, and striatum. Hypoactivation in the right angular gyrus was positively correlated with the illness duration of BD. The present study reveals the potential neural mechanism underlying impairment in reward anticipation in BD. Some clinical features such as clinical subtype, mood state, and duration of illness confound the underlying neurobiological abnormality reward anticipation in BD. These findings may have implications for identifying clinically relevant biomarkers to guide intervention strategies for BD.

## Introduction

Bipolar disorder (BD) is a severe psychiatric disorder, characterized by depressive, manic, and mixed episodes with variable inter-episode remission. Individuals with BD often show excessive goal-directed and pleasure-seeking behavior during manic episodes while decreased hedonic capacity during depressive episodes, together with strong desires for goals and reward even in remission [1–3], suggesting impaired reward processing throughout BD. Moreover, altered reward processing is associated with the severity of clinical symptoms in BD [4, 5], and influences the development and course of BD [6–8]. According to the Behavioral Approach System (BAS) dysregulation model, reward hypersensitivity is related to both hypomanic/manic and depressive symptoms in individuals with BD when they respond to reward-relevant events [9, 10], and remains elevated in remission [11–13]. Hypersensitivity to reward-relevant stimuli may be a key component of emotional dysregulation, vulnerability, and affective lability in BD [14–16]. Exploring the neurobiological basis of impairments in reward processing in individuals with BD may thus be helpful to improve treatment and prevention.

Clinical research works have reported altered anticipatory processing in BD, which results in abnormalities in assigning the motivational value to anticipated outcomes and impaired decision-making strategies [17–19]. Reward anticipation is the initial prospect of a reward encountered during reward processing [20, 21], which motivates individuals to produce incentive motivation and make efforts to achieve goals [22]. In healthy individuals, reward anticipation processing such as signaling about anticipated reward levels [21, 23], activating under anticipated arousal and effort [24, 25], and processing outcome predictability [26–28] depends on the function of the ventral striatum (VS), the anterior cingulate cortex (ACC), and the parietal regions, respectively. Existing studies have proposed that aberrant responses in some reward-related brain regions, such as the ACC, the orbitofrontal cortex (OFC), and the striatum, confer risk for the development of bipolar spectrum disorders [29, 30]. Individuals with BD present abnormal activation of the cortical-striatal circuit during the performance of reward-relevant tasks [31–33]. For example, some whole-brain studies found hyperactivation in the prefrontal and cingulate cortex in euthymic individuals with BD during reward anticipation [34–37], while others found hypoactivation in the parietal lobe [31, 32].

The VS has been implicated as a key area coding reward anticipation [38, 39], which encompasses the ventral part of the caudate and the nucleus accumbens, and receives projections from dopaminergic cells respond to reward-predicting cues and top-down regulation from cortical regions [40–42]. An electroencephalography study provides evidence of a top-down regulation from the frontal area to VS during reward anticipation [40]. It is clear that the VS participates in several extended networks linked to a range of cognitive, affective, and social behaviors [43]. And resting-state functional connectivity study has reported this circuit-level alteration, which suggested attenuation of functional connectivity between the OFC and VS in BD [44]. However, a mixed pattern of VS activation was reported in response to reward in BD, with hyperactivation in the VS during reward anticipation in euthymic individuals with BD [34, 37] or hypoactivation of this region in manic individuals [45, 46]. From the above, there were no consistent findings regarding the neural activation alterations during reward anticipation in individuals with BD due to the small sample size, the heterogeneity in sample characteristics, and methodology. The heterogeneity reminds the necessity of further identifying specific neural mechanisms of reward anticipation in BD.

The primary aim of the current study was to explore the neural basis underlying impairment of reward anticipation in BD by whole-brain- and specific VS ROI-based meta-analyses. Furthermore, separate analyses were performed for individuals with different sub-types and clinical states of BD. Finally, the potential effects of clinical features on functional activation in BD were investigated using meta-regression analysis.

## Materials and methods

### Search strategies

A literature search of PubMed, Embase, ScienceDirect, and Web of Science databases was conducted to identify original fMRI studies of BD individuals performing reward-relevant tasks, which had been published in the English language in peer-reviewed journals up to June 2021. The search strategy included different combinations of the following terms: (‘bipolar disorder’ OR ‘manic depressive psychos*’ OR ‘mani*’ OR ‘bipolar depression’ OR ‘bipolar affective psychos*’) AND (reward* OR ‘risk’ OR ‘risk taking’) AND (‘magnetic resonance imaging’ OR MRI OR fMRI OR ‘functional magnetic resonance imaging’). The retrieved articles, including relevant reviews and meta-analyses, were searched to identify original studies that were potentially missed in the above searches.

### Selection criteria

An fMRI study was retained if (1) a precise diagnosis of BD was made, (2) brain activation during reward anticipation was compared between individuals with BD and HC, (3) individuals were equal to or over 18 years old, (4) whole-brain and/or ROI analysis was used, and (5) stereotactic 3D coordinates of brain activation were reported.

Studies were excluded if (1) results were not based on the main effects of the group, (2) subjects were under 18 years old, (3) only small volume correction was used, and (4) the peak coordinates of effects were unavailable even after the authors were contacted via e-mail. If studies reported longitudinal experiments, only the baseline results were included.

### Data extraction and quality assessment

This meta-analysis followed the guidelines for a Meta-analysis of Observational Studies in Epidemiology (MOOSE) (Supplementary Table S1). The following information was compiled for all the included studies: first author, year of publication, cohort size, age, sex, age at onset, illness duration, illness subtype, mood state, Hamilton Depression Scale (HAMD), Young Manic Rating Scale (YMRS), comorbidity, medication, task paradigm, imaging parameters (slice thickness, magnetic field strength, smoothing kernel, stereotactic template space, analysis software), and statistical threshold.

The peak coordinates and corresponding *t* statistics of significant differences in brain activation were extracted into a text file for each study. The results of studies using whole-brain and ROI-based analyses were retrieved and summarized, respectively. The literature search and data extraction were independently conducted by two authors (XPL and XLW). When extra information was required, a request was made to the corresponding author by e-mail.

In addition, a quality assessment score was computed according to the criteria modified from studies by Sanderson et al. [47] and Shepherd et al. [48]. The relevant checklist included 15 items relating to, for example, demographics, method of recruitment, task design, image acquisition and analysis, and consistency of the conclusions (Supplementary Table S2).

### Coordinate-based meta-analysis

Anisotropic Effect-Size version of Seed-based Signed Differential Mapping (AES-SDM) software [49, 50], version 5.15 (https://www.sdmproject.com/), was used to investigate brain regions that potentially show consistent significant differences in brain activation between BD individuals and controls during reward anticipation.

#### Main meta-analysis

For the whole-brain meta-analysis, effect-size maps of differences between groups for each study were recreated to generate Monte Carlo brain maps by randomly permuting voxels from these brain maps. Then, estimated statistical maps were included in a random-effect meta-analytic model that weighted the contribution of each study according to its sample size. The threshold was set at *p* < 0.005 (voxel level), with SDM-*z* > 1 (peak height) and a cluster size ≥10 voxels, since it was found to be optimally balanced sensitivity and specificity in AES-SDM meta-analysis and was adopted by most of the previous AES-SDM studies [49, 51, 52].

For the ROI meta-analysis, we selected bilateral VS (including nucleus accumbens) as the ROIs, which were defined based on the Harvard-Oxford subcortical atlas [53]. We examined activation differences in left and right VS between BD and HC at *p* < 0.005 separately.

#### Subgroup meta-analysis

Subgroup meta-analyses were performed for studies that recruited individuals with BD type I (BD I) and individuals with euthymic BD, respectively.

#### Reliability analysis

In order to assess the effect of an individual study on the estimated pooled effect size, a whole-brain jack-knife sensitivity analysis was performed in which each study was discarded at successive repeat iterations in the meta-analysis.

#### Heterogeneity and publication bias analyses

The statistical heterogeneity of individual clusters was examined using a random-effect model with Q statistics (*X*^2^) distribution converted to *z* values and tested with a permutation approach (*p* < 0.005, uncorrected; peak height *z* = 1; cluster extent = 10). Publication bias was evaluated using Egger’s test (*p* < 0.05) [54].

#### Meta-regression analysis

The potential effects of average age, male percentage, age at onset, illness duration, HAMD score, YMRS score in the BD cohort, percentage of medication-free individuals, and quality score on the results were explored by meta-regression using a linear random-effect model. As in previous meta-analysis, in order to minimize the detection of spurious relationships, a threshold of *p* < 0.0005 was used and only brain regions with significant results in the main meta-analysis were considered [50, 52].

## Results

### Sample characteristics of studies included in the whole-brain meta-analysis

**Fig. 1:**
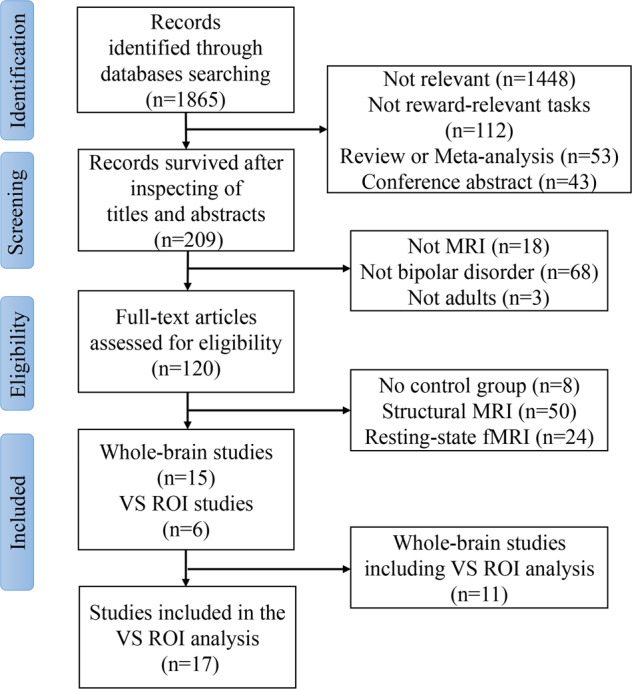
Flow diagram of the literature search in this meta-analysis. ROI region of interest, VS ventral striatum.

Fifteen studies (including 16 experiments) met the inclusion criteria for whole-brain-based meta-analysis, with a total of 372 BD individuals and 507 HC (Fig. 1). There were no significant differences in mean age between individuals with BD and HC (BD: 36.37 ± 9.73 years, HC: 34.76 ± 7.78 years, *t* = 0.60, *p* = 0.56). BD individuals showed a lower percentage of males than HC (BD: 159/372 = 42.74%, HC: 259/507 = 51.08%, *χ*^2^ = 5.99, *p* < 0.05).

The paradigms of reward-relevant tasks included Monetary Incentive Delay (eight studies), Card Guessing (five studies), Social Incentive Delay (three studies), and Lowa Gambling (one study). Twelve studies reported no significant difference in task performance or reaction time between BD individuals and HC, with three studies [32, 33, 55] providing no relevant information. The quality assessment scores ranged from 11.5 to 15 points with an average of 13.4 points. More detailed information about the demographic and clinical characteristics and the quality assessment score of all included studies are presented in Table 1. The details of the imaging parameters, statistical threshold, and results of the between-group analysis for each study are presented in Supplementary Table S3.

**Table 1:** Demographic and clinical characteristics of all individuals included in the meta-analysis.

Author, year	Sample size	Male/female		Age (year)	Age at onset (year)	Illness duration (year)	Subtype	Mood state	Scale	Type of reward	NMF	PC	QS
	P/HC	P	HC	P/HC									
*Whole-brain studies*													
Anna Manelis, 2018	34/17	5/29	7/10	35.07/31.41	19.06	16.01	BD I	Dep	2.79 (YMRS) 14.35 (HAMD)	Card guessing	0	NA	13.5
Anup Sharma, 2016	24/38	10/14	17/11	38/39.4	NA	15.3	BD I BD II	Dep	NA	Social reward	0	NA	11.5
Claudia Hagele, 2015	13/54	8/5	41/13	36/37.7	NA	12.3	BD I	Mania	19.6 (YMRS)	Monetary incentive delay task	1 (8%)	No	14
Felix Bermpohl, 2010	15/26	8/7	15/11	38.6/38.7	NA	16.2	BD I	Mania	18.9 (YMRS)	Monetary incentive delay task	0	No	12
Henry W. Chase, 2013	23/37	4/19	12/15	33.94/33.09	16.65	17.29	BD I	Dep	4.0 (YMRS) 24.7 (HAMD)	Card guessing	0	Yes	14
Jigar Jogia, 2012	36/37	17/19	21/16	42.5/37.6	22.4	20.1	BD I	Eut	1.7 (YMRS) 1.8 (HAMD)	Lowa gambling task	14 (39%)	NA	14
Kristina Schwarz, 2020	28/110	12/16	54/56	34/30.4	NA	NA	BD I	NA	2.8 (YMRS) 8.0 (HAMD)	Monetary incentive delay task Social reward task	0	NA	12.5
Matthias Krischner, 2019	25/25	16/9	16/9	37.3/33.1	21.9	15.4	BD I	Eut	4.7 (HAMD)	Monetary incentive delay task	0	No	15
Robin Nusslock, 2012	21/20	9/12	8/12	31.53/31.56	18.14	13.39	BD I	Eut	2.29 (YMRS) 6.43 (HAMD)	Card guessing	20 (95%)	Yes	14
Ronny Redlich, 2015	33/34	17/16	16/17	38.12/38.59	NA	11.54	BD I	Dep	2.45 (YMRS) 22.88 (HAMD)	Card guessing	0	Yes	13
Sarah W. Yip, 2015	20/20	12/8	10/10	22.59/22.1	NA	NA	BD II NOS	Dep	1.24 (YMRS) 9.24 (HAMD)	Monetary incentive delay task	20 (100%)	Yes	13.5
Sheri L. Johnson, 2019	24/24	12/12	13/11	37.04/33.92	NA	NA	BD I	Eut	4.03 (HAMD)	Monetary incentive delay task	24 (100%)	No	13.5
Sunny J. Dutra, 2015	24/25	9/15	10/15	31.38/29.44	16.33	14.78	BD I	Eut	1.5 (YMRS)	Monetary incentive delay task Social incentive delay task	0	Yes	14.5
Stefanie Schreiter, 2016	20/20	8/12	12/8	41.6/41.45	26.85	15.5	BD I BD II	Eut	0.5 (YMRS) 1.85 (HAMD)	Monetary incentive delay task	0	No	12
Xavier Caseras, 2013	17/20 15/20	6/11 6/9	7/13	42.82/42.30 40.53/42.30	17.57 18.92	NA	BD I BD II	Eut	3.17/1.80 (YMRS) 3.88/2.67 (HAMD)	Card guessing	0	Yes	13.5
*ROI studies*													
Bianca Kollmann, 2017	16/24	6/10	12/12	43.13/42.73	25.63	NA	BD I	Eut	0.19 (YMRS) 1.13 (HAMD)	Monetary incentive delay task	16 (100%)	No	10.5
Birgit Abler, 2008	12/12	7/7	NA	33.9/36.2	NA	12.8	BD I	Mania	21.8 (YMRS)	Monetary incentive delay task	0	NA	11
Jason Smucny, 2021	22/49	15/7	33/16	21.4/20.2	NA	0.72	BD I	NA	3.2 (YMRS)	Incentivized Control Engagement	4(18%)	NA	9
Julia Linke, 2012	19/22	8/11	11/11	45/28	29.6	NA	BD I	Eut	0.9 (YMRS) 1.0 (HAMD)	Probabilistic reversal learning task	0	NA	11
Lisa H. Berghorst, 2016	13/15	5/8	5/10	27.01/31.73	NA	NA	BD I BD II	NA	3.08 (YMRS) 5.62 (HAMD)	Monetary incentive delay task	0	Yes	14
Sarah Trost, 2014	16/16	6/10	7/9	35.6/35.4	24.5	11.1	BD I	NA	2.3 (YMRS)	Desire-reason dilemma task	0	Yes	11

*BD* bipolar disorder, *Dep* depression, *Eut* euthymic, *HAMD* Hamilton Depression Scale, *HC* healthy controls, *NMF* number of patients of medication free, *P* patients, *PC* psychiatric comorbidity, *QS* quality score, *ROI* region of interest, *YMRS* Young Manic Rating Scale.

### Whole-brain meta-analysis

#### Main meta-analysis

**Fig. 2:**
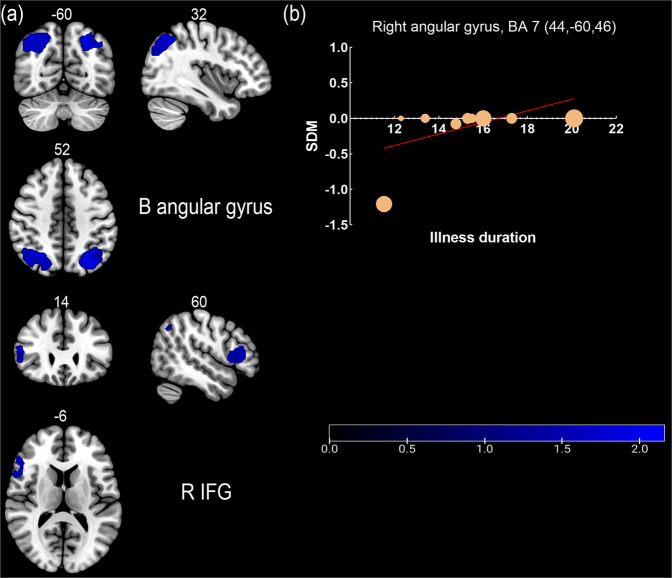
Results of whole-brain meta-analysis and meta-regression analysis. **a** Results of meta-analysis showed that BD individuals showed significant hypoactivation in the bilateral angular gyrus and the right inferior frontal gyrus. **b** Illness duration of individuals with BD is positively associated with activation of the right angular gyrus. Blue clusters represent hypoactivation in individuals with BD compared to healthy controls. B bilateral, BA brodmann area, IFG inferior frontal gyrus, R right.

Compared with HC, BD individuals showed significant hypoactivation in the bilateral angular gyrus and right inferior frontal gyrus, with no brain region showing significant hyperactivation (Table 2 and Fig. 2a).

**Table 2:** Significant differences in brain activation between individuals with BD and HC.

	Maximum				Clusters
Brain regions (peak)	MNI coordinate *x*, *y*, *z*	SDM *z*-value	*P* value uncorr	No. of voxels	Breakdowns (no. of voxels)
BD > HC					
None					
BD < HC					
R angular gyrus, BA 7	32, –60, 52	–2.147	0.000005960	1709	R angular gyrus, BA 39 (255)
					R superior parietal gyrus, BA 7 (250)
					R angular gyrus, BA 7 (249)
					R middle occipital gyrus, BA 19 (176)
					R superior occipital gyrus, BA 7 (152)
					R superior occipital gyrus, BA 19 (123)
L angular gyrus, BA 7	–38, –72, 42	–2.190	0.000003219	1072	L superior parietal gyrus, BA 7 (253)
					L inferior parietal gyrus, BA 7 (233)
					L middle occipital gyrus, BA 19 (123)
R inferior frontal gyrus, BA 48	60, 14, 6	–1.690	0.000495315	488	R inferior frontal gyrus, BA 45 (240)
					R inferior frontal gyrus, BA 48 (110)

*BA* Brodmann area, *BD* bipolar disorder, *HC* healthy controls, *L* left, *MNI* Montreal Neurological Institute, *R* right, *SDM* seed-based d mapping.

#### Subgroup meta-analyses

The first subgroup analysis including 11 studies observed hyperactivation in the left anterior cingulate gyrus, and hypoactivation in the left middle occipital gyrus, right inferior frontal gyrus, and right angular gyrus in individuals with BD I compared to HC (Table 2 and Fig. 2b). The second subgroup analysis including eight studies observed hyperactivation in the left orbital frontal gyrus, left fusiform gyrus and left insula, and hypoactivation in the right inferior temporal gyrus and right striatum in individuals with euthymic BD compared to HC (Table 2 and Fig. 2c).

#### Reliability analyses

A whole-brain jack-knife sensitivity analysis of the main meta-analysis showed that hypoactivation of the bilateral angular gyrus was preserved in all 16 datasets, and the right inferior frontal gyrus remains significant in fourteen datasets (Supplementary Table S5).

#### Heterogeneity and publication bias analyses

There was no significant between-study heterogeneity in the results for the main and both subgroup meta-analyses. None of the clusters reported above showed significant publication bias based on Egger’s test (*p* > 0.05) in the main meta-analysis.

#### Meta-regression analyses

**Fig. 3:**
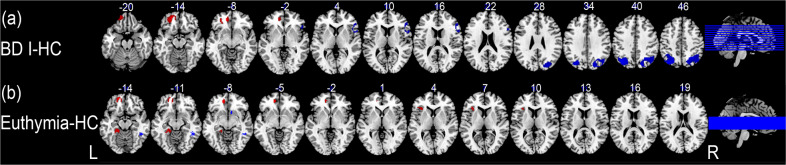
Subgroup analyses of BD I and euthymic BD individuals. **a** Hyperactivation in left ACC and hypoactivation in the right angular gyrus, left middle occipital gyrus, and right inferior frontal gyrus were found in individuals with BD I relative to HC. **b** hyperactivation in the left OFC, left fusiform gyrus and insula, and hypoactivation in right temporal gyrus and striatum were found in individuals with euthymic BD relative to HC. Red and blue clusters represent hyperactivation and hypoactivation in individuals with BD compared to healthy controls, respectively. ACC anterior cingulate cortex, BD bipolar disorder, HC healthy controls, L left, OFC orbital frontal cortex, R right.

The meta-regression analyses showed that the illness duration of BD individuals was positively correlated with hypoactivation in the right angular gyrus (MNI coordinates: *x* = 44, *y* = –60, *z* = 46; 19 voxels; SDM = –4.244; *p* < 0.001; Fig. 3). The meta-regression analysis showed no effect related to the average age, age at onset, HAMD score, YMRS score of the patient cohort, percentage of medication free individuals, or quality assessment score.

### Sample characteristics of studies included in the ROI meta-analysis

Seventeen studies (eleven from whole-brain studies) comprising 18 samples were included in the VS ROI meta-analysis, with a total of 348 individuals with BD and 443 HC (Fig. 1). There were no significant differences in mean age (BD: 32.85 ± 6.51, HC: 33.44 ± 6.26, *t* = 0.68, *p* = 0.49) or male percentage (BD: 162/348 = 44.11%, HC: 204/443 = 51.08%, *χ*^2^ = 0.02, *p* = 0.89) between individuals with BD and HC.

The paradigms of reward-relevant tasks included Monetary Incentive Delay (ten studies), Card Guessing (four studies), Probabilistic Reversal Learning (one study), Social Incentive Delay (one study), and Incentivized Control Engagement (one study). Nine studies reported no significant difference in task performance or reaction time between BD individuals and HC, with one study providing no relevant information [55]. Four studies reported no significant difference in task performance between BD individuals and HC, and three studies reported lower accuracy [45, 46] and longer reaction times [56] in task performance in BD individuals than HC. The quality assessment scores ranged from 9 to 14 points with an average of 11.2 points. More detailed information about the demographic and clinical characteristics and the quality assessment score of all included studies are presented in Table 1. The details of the imaging parameters, statistical threshold, and results of the between-group analysis for each study are presented in Supplementary Table S3.

### ROI-based meta-analysis

No significant activation difference was observed for bilateral VS ROIs between BD individuals and HC (effect size for left VS: 0.96; effect size for right VS: 0.97; *p* > 0.005).

## Discussion

The current study revealed hypoactivation during reward anticipation in the bilateral angular gyrus and right inferior frontal gyrus in individuals with BD relative to HC by whole-brain meta-analysis, with no significant activation abnormality in bilateral VS by whole-brain or ROI analysis. Anomalous functioning of the frontal-parietal is the most significant finding in BD individuals. Hypoactivation in the right angular gyrus was positively correlated with illness duration in individuals with BD. Certain clinical characteristics such as illness subtype, mood state, and duration of illness influenced the brain activation alterations produced by BD during reward anticipation.

### Hypoactivation of the prefrontal-parietal regions during reward anticipation

Our findings are consistent with other studies which reported hypoactivation in the right inferior frontal gyrus during reward processing by positron emission tomography [57], and decreased activity in the prefrontal cortex during Iowa Gambling Task by near-infrared spectroscopy [58] in individuals with BD relative to HC. Altered activation in the inferior frontal gyrus, an important part of the frontal-striatal circuit, is associated with reward hypersensitivity in BD [29, 30]. The inferior frontal gyrus is an important part of the lateral prefrontal cortex, which upregulates activity in the limbic and mesolimbic systems [59, 60]. Dysfunction of the inferior frontal gyrus has been observed in individuals with BD during executive control [61–63], and reward signals play a crucial role during this process [64–66]. The angular gyrus, a cross-modal integrative hub that converges multisensory information, can detect discrepancies between predicted and actual action consequences for multimodal feedback [67]. Activation in the angular gyrus during reward anticipation was correlated with nucleus accumbens dopamine release [68], which supports the hyperdopaminergia theory across mood states in BD [6]. The hyperdopaminergia theory suggests that an increase in striatal dopamine transporter levels may lead to a decrease in dopaminergic function and depression [69], while an increase in striatal dopamine receptor levels may lead to an increase in dopaminergic neurotransmission and mania [70, 71]. The disturbance in dopamine system homeostasis may be one of the pathophysiologies of BD, which has been tested to be a close connection with reward processing [72]. We also observed a positive correlation between hypoactivation of the angular gyrus and illness duration in BD individuals, which may suggest gradually impaired reward function during the development of illness [7].

The inferior frontal gyrus and angular gyrus are key components in the executive control network, which is assumed to modulate reward systems [73–75]. An intact executive functioning network may dampen an overactive reward system and therefore promote adaptive functioning [76]. Higher executive functioning was associated with increased activation in parietal areas during reward anticipation and increased limbic connectivity with frontal areas [77]. Decreased engagement of prefrontal-parietal regions may reflect difficulties in inhibiting excessive pleasure-seeking, increased impulsive behavior, and pronounced risk-taking tendencies in BD [78, 79].

### Subgroup findings in BD

Individuals with BD I showed hyperactivation in the left dorsal ACC, and hypoactivation in the left middle occipital gyrus, right inferior frontal gyrus, and angular gyrus compared to HC. The ACC receives projections from the OFC, VS, and mesolimbic dopamine system, and is implicated in risk decision and uncertainty assessment [24, 80, 81]. The dorsal ACC plays a critical role in forming associations between rewards and actions [82]. Hyperactivation in the dorsal ACC may result in excessive stimulation of mesolimbic dopamine release, manifested as exaggerated hedonic responses and enhanced motivational drive [83]. Abnormalities of the occipital gyrus have been observed in spontaneous neural activities and emotional processing in BD [84–86]. The frontal-striatal and occipital regions are reliably activated during reward anticipation [87, 88]. Our results add to the evidence of functional impairment during reward anticipation in prefrontal and parietal-occipital regions in BD I.

Euthymic BD also showed hyperactivation in the left OFC, fusiform gyrus and insula, and hypoactivation in the right inferior temporal gyrus and striatum during reward anticipation. The OFC involves the first stage of cortical processing that represents reward value [89], and activation in this region updates rapidly when reward value changes and sends this information to the ACC for actions guided by outcomes [90]. The ventral temporal cortex is a key structure in high-level visual processing [91–93] and represents objects independently of their reward value [94]. Activation of the insula is correlated with subjective affective experience of rewards since the insula plays an important role in interoception [95]. The frontal-striatal circuit is a well-established neural pathway in the reward system, which involves dopaminergic projection from the midbrain nuclei to subcortical areas that are central to processing the reward properties of stimuli, and to cortical targets [96]. The nucleus accumbens, which is the center of VS and receives projections from the OFC, ACC, amygdala, and midbrain, can integrate incoming dopaminergic signals from cortical and limbic regions to guide decision making, track the outcomes of actions, and influence the direction of future ones [97]. These regions are involved in the valuation/motivation network [98] and salience/monitoring network [99, 100], which play an important role during reward anticipation in BD. These findings provide evidence that abnormal brain activation remains in frontal-temporal-striatal regions during reward anticipation in euthymic BD.

### Null findings in VS

Despite negative findings during reward anticipation in BD by our whole-brain and ROI analyses, the VS has a relatively specific role in reward processing compared to other cognitive processes [101, 102]. It seems premature to draw the conclusion of no abnormal activation in VS in BD during reward processing, especially in reward anticipation. First, the heterogeneity of individuals in the included studies may bias the findings. For example, our subgroup analysis found significant hypoactivation in VS in individuals with euthymic BD. According to the BAS/reward hypersensitivity model of BD, the VS has been engaged contingent on mood, whereas elevated mood may increase the expected value and elicit VS activity but low mood may decrease the perceived value and dampen VS activity [103]. Second, the different processing types of the tasks also affected brain activation. Besides reward anticipation, VS was also found to have altered activation in reward receipt and loss anticipation in BD [12, 37, 104–106]. Moreover, striatal activation to reward cues is modulated by several factors involved in reward anticipation including the magnitude of the reward, the probability of reward receipt, the amount of time until the anticipated reward can be obtained, and the effort required to pursue the reward [107]. These studies suggest a complex role of VS in reward processing, suggesting the need for sophisticated fMRI protocols to separate them at the brain level.

In addition, reward deficits in mood disorders were associated with altered connectivity between VS and large-scale functional networks [108]. Particularly, reward anticipation was characterized by dense connectivity in the frontal-parietal-temporal-striatal network in BD [109]. Some studies have shown that individuals with BD exhibited decreased dynamic functional connectivity in frontal areas and increased VS and OFC functional connectivity during reward processing [110, 111]. In this context, altered activation in a selected region cannot fully explain complex patterns of reward impairments in BD; therefore, it is important to examine the functioning at the level of the brain network in future studies.

### Limitations

This meta-analysis has several limitations that should be acknowledged. First, the number of studies included in the meta-analyses was relatively small, which limited the statistical power, especially in the case of different clinical sub-types and mood states of BD. Second, the comparability of different task paradigms’ difficulty and discriminability is an important question that awaits further work. Third, this study focused on the brain activation alterations of the reward anticipation phase during reward processing in BD. Thus, the results do not represent the full range of reward processing. It will be an interesting topic to discover whether a functional abnormality in a particular brain region may underlie impairment in loss anticipation, reward/loss outcome, and prediction error in individuals with BD. Fourth, only VS ROI meta-analysis was performed in BD due to the limited number of studies. Finally, the possible effect of medication on the findings in BD cannot be totally ruled out. The potentially confounding effects of psychotropic medication in bipolar neuroimaging research have been discussed previously, which found no or limited impact on fMRI results [112, 113]. However, we cannot completely rule out specific medication effects considering the evidence that psychotropic medications might generally blunt neural responses to reward anticipation [34, 35]. Further studies recruiting unmedicated patients or studies with a longitudinal design controlling for medication are needed.

## Conclusions

The present study revealed significantly altered brain activation in prefrontal and inferior parietal lobule regions during reward anticipation processing in BD, suggesting the potential neurobiological mechanism underlying impairment in reward anticipation in BD. The clinical features of individuals with BD may affect the neurobiological basis during reward anticipation. Future prospective studies, recruiting different subgroups of BD, focusing on other phases of reward processing such as loss anticipation, reward outcome and prediction error, and using multimodal neuroimaging, are needed to better understand the longitudinal neural trajectory underlying reward processing in BD.

## Supplementary information

Supplementary material
