# Supplementary material for: Altered brain activation during reward anticipation in bipolar disorder
Source: Transl Psychiatry. 2022 Jul 28;12:300. doi: 10.1038/s41398-022-02075-w (PMC9334601; doi:10.1038/s41398-022-02075-w)
Supplement: Supplementary file 1 — Supplementary material [file 41398_2022_2075_MOESM1_ESM.doc]

**Supplementary materials**

**Table S1** MOOSE checklist.

**Table S2** Quality assessment checklists (Score 0/0.5/1 for each item *, total score 15 out of 15).

**Table S3** Imaging parameters, statistical threshold and the results of between-group analysis for each research.

**Table S4** Subgroup analyses in individuals with BD type I and individuals euthymic BD.

**Table S5** Sensitivity analysis.

**Table S1** MOOSE checklist

| **Criteria** | | **Brief description of how we handled the criteria in the meta-analysis** | |
| --- | --- | --- | --- |
| **Reporting of** **background should include** | | | |
| Problem definition | | A number of studies have used functional MRI to identify reward-related dysfunction in individuals with bipolar disorder (BD), but the findings were disparate. | |
| Hypothesis statement | | First, BD individuals showed abnormalities in reward processing and approach-related affect, which plays an important role in the pathophysiology of BD. Second, BD individuals showed the most reliable and consistent pattern of brain functional alterations in frontal-striatal systems. | |
| Description of study outcomes | | Frontal-striatal systems are thought to be the neural basis of reward-related abnormalities. | |
| Type of exposure or intervention used | | Functional magnetic resonance imaging (fMRI) scan was performed while both BD individuals and controls were conducting reward-related tasks. | |
| Type of study designs used | | Cross-sectional fMRI studies. | |
| Study population | | Subjects with ICD/DSM diagnosis of bipolar disorder and matched healthy controls. | |
| **Reporting of search strategy should include** | | |  |
| Qualifications of searchers | | Investigator (Long) and is a candidate for Ph.D. and investigator (Wang) is M.D. Long has attended a meta-analysis training class hold by the Chinese Cochrane Center. | |
| Search strategy, including time period included in the synthesis and keywords | | Pubmed, Embase, ScienceDirect and Web of Science up to June 2021, with the search strategy (“bipolar disorder” OR “manic depressive psychos*” OR mani* OR “bipolar depression” OR “bipolar affective psychos*”) AND (“reward" OR "risk " OR "risk taking") AND (fMRI OR “functional magnetic resonance imaging”) | |
| Databases and registries searched | | • PUBMED (Jan 2021 – June 2021) = 436 hits  • EMBASE (Jan 2021 – June 2021) = 276 hits  • ScienceDirect (Jan 2021 – June 2021) = 868 hits  • Web of Science Core Collection (Jan 2021 – June 2021) = 245 hits | |
| Search software used, name and version, including special features | | • PubMed was accessed from the US National Library of Medicine National Institutes of Health • Embase are available on the OVID SP platform • ScienceDirect belongs to Elsevier  • Web of Science Core Collection is part of Web of Science | |
| Use of hand searching | | Hand searching was used in previous related reviews | |
| List of citations located and those excluded, including justifications | | Details of the literature search processed are presented in Figure 1. | |
| Method of addressing articles published in languages other than English | | Only the articles written in English were included. | |
| Method of handling abstracts and unpublished studies | | Abstracts were excluded, we did not search for unpublished studies | |
| Description of any contact with authors | | We contacted the corresponding author when necessary data is missing. | |
| **Reporting of methods should include** | | | |
| Description of relevance or appropriateness of studies assembled for assessing the hypothesis to be tested | | We have described the inclusion and exclusion criteria in the Method section | |
| Rationale for the selection and coding of data | | Data we extracted were related to the population characteristics, study design, image acquisition and analysis and possible confounders. | |
| Assessment of confounding | | Meta-regressions were used to examine the influence of mean age, male percentage, age at onset, duration of illness, HAMD score, YMRS score, quality score and smooth kernel. Subgroup analyses focus on the non-social reward tasks, depressive individuals, euthymic individuals and individuals diagnosed as BD I. | |
| Assessment of study quality, including blinding of quality assessors; stratification or regression on possible predictors of study results | | We assessed the study quality through four categories: subjects, methods for reward-related tasks, methods for image acquisition and statistical analysis, Results, conclusions and conflict of interest. | |
| Assessment of heterogeneity | | Heterogeneity across studies was investigated with meta-regression and by checking the map of Q_H_ statistics. | |
| Description of statistical methods in sufficient detail to be replicated | | We described the software corresponding web link and the critical steps used to fulfil this meta-analysis. | |
| Provision of appropriate tables and graphics | | We provided two tables to describe study characteristics and main results of this meta-analysis. Two sections were used to describe the main findings of the analyses. | |
| **Reporting of results should include** | | | |
| Graph summarizing individual study estimates and overall estimate | It was implemented in the main text. | | |
| Table giving descriptive information for each study included | It was implemented in the main text. | | |
| Results of sensitivity testing | We reported the results of sensitivity testing according to Jackknife analysis. | | |
| Indication of statistical uncertainty of findings | Effect size and P-values. | | |
| **Reporting of discussion should include** | | | |
| Quantitative assessment of bias | We quantitatively assess the publication bias by Egger's test. | | |
| Justification for exclusion | The exclusion criteria were in the *Methods* section. | | |
| Assessment of quality of included studies | We perform a quality assessment based on previous meta-analysis and revise it according to the condition of our meta-analysis. The detail can be found in the Table S2. | | |
| **Reporting of conclusions should include** | | | |
| Consideration of alternative explanations for observed results | See Discussion | | |
| Generalization of the conclusions | See *Conclusion*. | | |
| Guidelines for future research | See *Discussion*. | | |
| Disclosure of funding source | See *Acknowledgements*. | | |

**Table S2 Quality assessment checklists (Score 0/0.5/1 for each item*, total score 15 out of 15).**

| **Category 1: Subjects** |
| --- |
| 1. Patients were evaluated prospectively, specific diagnostic criteria were applied, and demographic data was reported |
| 1. Healthy comparison subjects were evaluated prospectively, psychiatric and medical illnesses were excluded and demographic data was reported |
| 1. Important variables (e.g. age, gender, intelligence quotient, i.e. IQ, handedness) were checked, either by stratification or statistically |
| 1. Withdrawals from the study were explained |
| **Category 2:** **Methods for** **executive function tasks** |
| 1. All participants went through a training session outside the scanner |
| 1. The baseline condition was defined as almost the same with task condition except for the reward control |
| **Category 3:** **Methods for image acquisition and statistical analysis** |
| 1. MRI slice-thickness ≤ 3 mm |
| 1. All images had < 2 mm movement |
| 1. The imaging technique used was clearly described so that it could be reproduced |
| 1. Adjustments were made for multiple statistical comparisons |
| 1. Appropriate design and/or analytical methods to control confounding |
| 1. Appropriate use of statistics for primary analysis of effect (excluding control of confounding) |
| **Category 4:** **Results, conclusions and conflict of interest** |
| 1. Statistical parameters for significant, and important non-significant, differences were provided |
| 1. Conclusions were consistent with the results obtained and the limitations were discussed |
| 1. Declarations of conflict of interest or identification of funding sources |
| *For criteria partially met, 0.5 points were given. |

**Table S3** Imaging parameters, statistical threshold and the results of between-group analysis for each research.

| **Study** | **Analysis software** | **Template** | **Magnetic field** | **Slice thickness, gap** | **Smoothing kernel** | **Threshold** | **ROI** | **BD>HC** | **HC>BD** |
| --- | --- | --- | --- | --- | --- | --- | --- | --- | --- |
| **Whole brain studies** | |  |  |  |  |  |  |  |  |
| Anna Manelis, 2018 | FSL | MNI | 3.0 T | NA | 6mm | *P_FWE_*<0.05 | - | - | - |
| Anup Sharma, 2016 | FSL | MNI | 1.5 T | 3.4mm, 0mm | 6mm | *P*_uncorr_<0.01, k>131 voxels | VS | - | - |
| Claudia Hagele, 2015 | SPM 8 | MNI | 1.5 T | NA | 8mm | *P_FWE_*<0.05 | VS/insula/amygdala | - | - |
| Felix Bermpohl, 2010 | SPM 5 | MNI | 1.5 T | NA | 8mm | *P_uncorr_*<0.001, k>5 voxels | VS/medial PFC/OFC | **Expected value:** left lateral OFC | **Cued incentive valence:** R PCC |
| Henry W Chase, 2013 | SPM 8 | MNI | 3.0 T | 3.1mm, | 5mm | *P*_uncorr_<0.005, k>10 voxels | ACC/vlPFC/VS | **Reward anticipation:** ACC/L SMA  **Anticipation per se:** L vlPFC |  |
| Jigar Jogia, 2012 | SPM 5 | Tal | 1.5 T | 3mm, 0.3mm | 8mm | *P_FWE_*<0.05 | - | **Reward anticipation:** L PCC/L ACC/ L mv PFC/ L parahippocampal gyrus | - |
| Kristina Schwarz, 2020 | SPM 12 | MNI | 3.0 T | 4mm, 1mm | 8mm | *P_FWE_*<0.05 | - | - | **Reward anticipation:** bilateral inferior parietal lobule, R lateral frontal gyrus |
| Matthias Krischner, 2019 | SPM 8 | MNI | 3.0 T | 3mm, 0.5mm | 6mm | *P_FWE_*<0.05 | VS/DS/IFG |  | **Reward anticipation:** fusiform gyrus, precentral gyrus, precuneus, lingual gyrus, superior parietal lobe |
| Robin Nusslock, 2012 | SPM 5 | MNI | 3.0 T | 1mm | 8mm | *P_uncorr_*<0.005, k>58 voxels | VS/OFC | **Reward anticipation:** L OFC/R VS/L VS/R OFC  **Loss anticipation:** R VS/L VS/R OFC |  |
| Ronny Redlich, 2015 | SPM 8 | MNI | 3.0 T | NA | 6mm | *P_uncorr_*<0.0005, k>79 voxels | Bilateral NAcc | - | **Reward anticipation:** bilateral NAcc/insula/IPG/MCC/MFG/Cuneus//Precentral gyrus |
| Sarah W Yip, 2015 | SPM 8 | MNI | 3.0 T | 4mm, 1mm | 6mm | *P_FWE_*<0.05 | VS/DS | - | **Reward anticipation:** R DS  **Loss anticipation:** R VS/R DS |
| Sheri L Johnson, 2019 | AFNI | Tal | 1.5 T | 4mm | 4mm | *P_corr_*<0.05 | Nucleus accumbens/MPFC | - | **High/medium gains:** nucleus accumbens  **Reward anticipation:** R nucleus accumbens R globus pallidus  **Gain outcomes:** L MOG |
| Sunny J Dutra, 2015 | FSL | MNI | 3.0 T | NA | 5mm | *P_corr_*<0.05 | VS/OFC | **Reward receipt**: R nucleus accumbens/R caudate/B thalamus | **Reward anticipation:** L OFC/R IFG/R LOC |
| Stefanie Schreiter, 2016 | SPM 8 | MNI | 3.0 T | 4mm, 1mm | 9mm | *P_uncorr_*<0.001, k>25 voxels | VS | - | **Reward anticipation:** bilateral VS |
| Xavier Caseras, 2013 | FSL | MNI | 3.0 T | 3.2mm | NA | *P_corr_*<0.05 | VS | **Reward anticipation:** STL/dl PFC/posterior insula/anterior insula/vl PFC/VS | - |
| **ROI studies** |  |  |  |  |  |  |  |  |  |
| Bianca Kollmann, 2017 | SPM8 | MNI | 3.0 T | 2.3mm, 0.7mm | 6.8*6.8*9mm | *P_FWE_*<0.05 | OFC/ACC /VS/insula | **Win anticipation:** Bilateral ACC |  |
| Birgit Abler, 2008 | SPM 2 | MNI | 3.0 T | 3mm, 0.75mm | 8mm | *P_FDR_*<0.005 | VS/tegmental area/brain stem |  | **Win vs. omission:** Nucleus accumbens |
| Jason Smucny, 2021 | SPM 8 | MNI | 3.0 T | NA | 8mm | *P_corr_*<0.05 | ACC/insula/VS/vl PFC | **Reward anticipation:** dorsal ACC | - |
| Julia Linke, 2012 | SPM 5 | MNI | 3.0 T | 2.3 mm, 0.7mm | 9mm | *P_FDR_*<0.05 | OFC /amygdala/ACC/striatum | **Win vs. baseline:** Medial OFC  **Reversal: lose/shift vs. baseline:** Medial OFC/ R OFC/R amygdala/dorsal ACC/putamen | - |
| Lisa H Berghorst, 2016 | FSL | MNI | 1.5 T | NA | 6mm | *P_corr_*<0.05 | Caudate/putamen/nucleus accumbens/amygdala | **Reward anticipation:** amygdala | **Reward anticipation under stress:** amygdala |
| Sarah Trost, 2014 | SPM 5 | MNI | 3.0 T | 3mm, 0.6mm | 9mm | *P_FWE_*<0.05 | Bilateral VS/VTA/ av PFC |  | **Reward anticipation/suppression of reward:** R VS/R thalamus/R MFG/R frontopercular cortex |

Abbreviations: ACC= anterior cingulate cortex; BD= bipolar disorder; VS= ventral striatum; DS= dorsal striatum; FSL: FMRIB Software Library; IFG= inferior frontal gyrus; IPC= inferior parietal cortex; L: left; LOC= lateral occipital cortex; MCC= middle cingulate cortex; MFG= middle frontal gyrus; MNI: Montreal Neurological Institute; MOG= middle occipital gyrus; MPFC= middle prefrontal cortex; NA: Not available, NAcc= nucleus accumbens; OFC= orbital frontal cortex; PCC= posterior cingulate cortex; PFC= prefrontal cortex; R: right; ROI= region of interest; SMA= supplementary motor area; SPM: Statistical Parametric Mapping, STL= superior temporal lobe; Tal: Talairach; VTA= ventral tegmental area; corr: corrected; dl=dorsal lateral; uncorr: uncorrected; vl= ventral lateral

**Table S4** Subgroup analyses in individuals with BD type I and euthymic individuals.

| **Brain regions** | **Maximum** | | | |
| --- | --- | --- | --- | --- |
|  | *MNI coordinate x, y, z* | *SDM value* | *P* value uncorrected | Number of voxels |
| **BD I** |  |  |  |  |
| ***BD>HC*** |  |  |  |  |
| L anterior cingulate cortex/L middle frontal gyrus, BA 11 | -14,44,-6 | 1.114 | 0.000051141 | 496 |
| ***BD<HC*** |  |  |  |  |
| R angular gyrus, BA 7/R superior parietal gyrus, BA 7/R middle occipital gyrus,BA 19/R superior occipital gyrus, BA 7 | 32,-58,52 | -2.178 | 0.000010788 | 1729 |
| L middle occipital gyrus, BA 19/L superior parietal gyrus, BA 7/L inferior parietal gyrus, BA7 | -38,-74,38 | -2.244 | 0.000005126 | 1108 |
| R inferior frontal gyrus, triangular part, BA 45 | 52,34,8 | -1.692 | 0.000749290 | 417 |
| **Euthymic** |  |  |  |  |
| ***BD>HC*** |  |  |  |  |
| L orbital frontal gyrus, BA 11 | -18,52,-14 | 1.264 | 0.000236213 | 230 |
| L fusiform gyrus, BA 30 | -22,-38,-14 | 1.013 | 0.002206922 | 176 |
| L insula, BA 48/47 | -32,26,6 | 1.045 | 0.001897991 | 61 |
| ***BD<HC*** |  |  |  |  |
| R inferior temporal gyrus, BA 20 | 46,-46,-10 | -1.005 | 0.002433717 | 75 |
| R striatum | 10,14,-8 | -1.003 | 0.002449989 | 20 |

Abbreviations: MNI: Montreal Neurological Institute, SDM: seed-based d mapping, L: left, R: right, BA: brodmann area.

**Table S5** Sensitivity analysis

| **Study** | **Decreased activations** | | |
| --- | --- | --- | --- |
|  | R angular gyrus | L angular gyrus | R inferior frontal gyrus |
| Bermpohl, 2010 | YES | YES | YES |
| Jogia, 2012 | YES | YES | YES |
| Nusslock, 2012 | YES | YES | YES |
| Caseras_BD I, 2013 | YES | YES | YES |
| Caseras_BD II, 2013 | YES | YES | YES |
| Chase, 2013 | YES | YES | YES |
| Dutra, 2015 | YES | YES | NO |
| Hagele, 2015 | YES | YES | YES |
| Redlich, 2015 | YES | YES | YES |
| Yip, 2015 | YES | YES | YES |
| Sharma, 2016 | YES | YES | YES |
| Manelis, 2018 | YES | YES | YES |
| Johnson, 2019 | YES | YES | YES |
| Krischner, 2019 | YES | YES | YES |
| Schwarz, 2020 | YES | YES | NO |
| Schreiter, 2016 | YES | YES | YES |

L: Left, R: Right.
